# How Well Does Evolution Explain Endogenous Retroviruses?—A Lakatosian Assessment

**DOI:** 10.3390/v14010014

**Published:** 2021-12-22

**Authors:** Ruben N. Jorritsma

**Affiliations:** Philosophy Group, Wageningen University & Research, 6700 EW Wageningen, The Netherlands; rubenjorritsma@gmail.com

**Keywords:** endogenous retroviruses (ERVs), endogenization-amplification theory (EAT), phylogenetics, evolution, methodology of scientific research programmes (MSRP), evolutionary research programme, long terminal repeats (LTRs)

## Abstract

One of the most sophisticated philosophies of science is the methodology of scientific research programmes (MSRP), developed by Imre Lakatos. According to MSRP, scientists are working within so-called research programmes, consisting of a hard core of fixed convictions and a flexible protective belt of auxiliary hypotheses. Anomalies are accommodated by changes to the protective belt that do not affect the hard core. Under MSRP, research programmes are appraised as ‘progressive’ if they successfully predict novel facts but are judged as ‘degenerative’ if they merely offer ad hoc solutions to anomalies. This paper applies these criteria to the evolutionary research programme as it has performed during half a century of ERV research. It describes the early history of the field and the emergence of the endogenization-amplification theory on the origins of retroviral-like sequences. It then discusses various predictions and postdictions that were generated by the programme, regarding orthologous ERVs in different species, the presence of target site duplications and the divergence of long terminal repeats, and appraises how the programme has dealt with data that did not conform to initial expectations. It is concluded that the evolutionary research programme has been progressive with regard to the issues here examined.

## 1. Introduction

### 1.1. The Methodology of Scientific Research Programmes

Two 20th century philosophers of science have strongly shaped how scientists, to this day, view what science is and how it works. The first was Karl Popper, who advocated falsifiability as the defining characteristic demarcating scientific theories from non-scientific ones [1]. The other was Thomas Kuhn, who held that in actual practice scientists are not attempting to falsify their most important theories—or paradigms—but are rather trying to preserve them [2]. Less well known is that the seemingly opposing views of Popper and Kuhn have been synthesized by the Hungarian philosopher Imre Lakatos [3]. This synthesis is called the methodology of scientific research programmes (MSRP).

**Figure 1:**
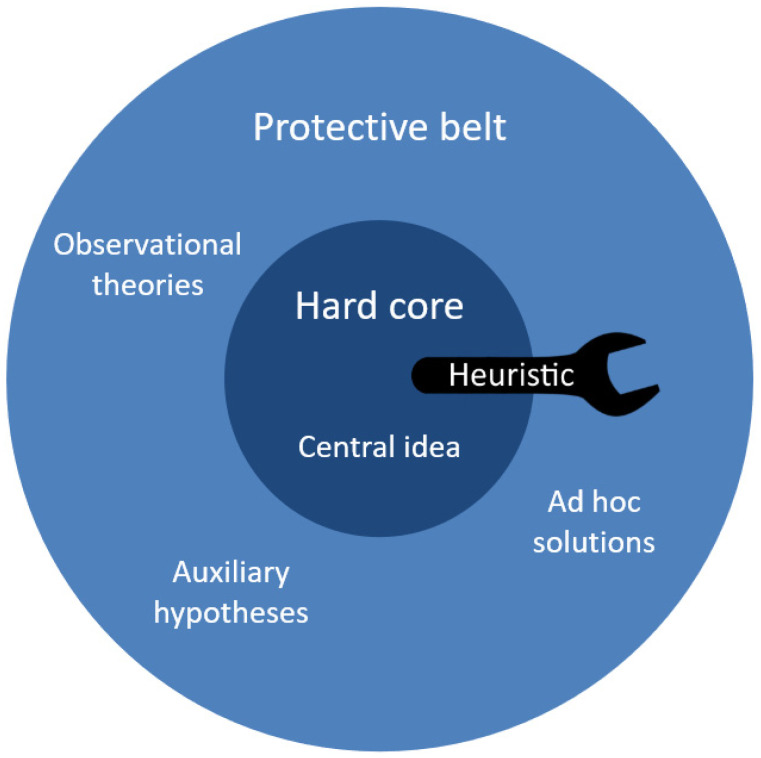
A research programme consists of a hard core of static beliefs, a heuristic, and a protective belt containing all other knowledge. The protective belt is dynamic and adapts in such a way that observations can be explained without affecting the hard core.

According to MSRP, scientists are working within so-called ‘research programmes’, which are somewhat similar to Kuhnian paradigms. A research programme consists of a static ‘hard core’ of fixed beliefs, and a dynamic ‘protective belt’ of auxiliary hypotheses and background knowledge (Figure 1). Whenever the programme is confronted with anomalous data, the hard core is shielded from refutation by changing something in the protective belt. There are innumerable ways in which the protective belt can be altered: this may involve the addition of a new parameter, different assumed starting conditions, the recognition of a new type of experimental error, or the proposal of a brand-new hypothesis—whatever is needed to account for the evidence without affecting the hard core.

In addition to a hard core and protective belt, a research programme also has a heuristic. This is a set of (mathematical or experimental) tools and principles that guides researchers as they develop the programme. The heuristic has a negative aspect (which simply says: preserve the hard core), but also a positive aspect. The positive heuristic tells researchers which questions to ask, where to look for interesting data, and how to sophisticate the protective belt in such a way that the programme as a whole can explain increasingly detailed data.

The Popperian element of MSRP surfaces in how Lakatos appraises research programmes. Programmes are assessed by how their protective belts evolve over time. When a research programme undergoes changes, whether it is in response to anomalies or by the forward momentum of the positive heuristic, Lakatos demands that these changes lead to the prediction of novel facts. If they do, the changes are ‘theoretically progressive’, and if the predictions are corroborated, they are ‘empirically progressive’, but if the changes merely accommodate already known facts in an ad hoc fashion, Lakatos calls them ‘degenerative’.

So rather than appraising theories in temporal isolation, under MSRP one must consider the progression of the research programme over an extensive period. If a research programme is characterized by progressive changes, scientists have good reason to continue scientific effort in that direction. If, on the other hand, a research programme generally features degenerative adjustments, scientists have a rational basis for switching to another, more promising research programme.

### 1.2. The Evolutionary Research Programme

For the past one and a half century, the field of biological origins has been dominated by the evolutionary research programme. The hard core of this programme says that all species have originated through natural processes by descent with modification from one or a few common ancestors.

Since this hard core was in place by the end of the 19th century, it is of interest to assess how well the programme has fared with the advances of molecular biology during the 20th and 21st centuries. Has the core thesis of large-scale common descent, combined with the heuristic tools of, for example, phylogenetics, led the way to the discovery of novel facts which may otherwise not have come to light? Or has the evolutionary programme merely provided some color commentary to the discoveries of molecular biologists, explaining their findings after the fact but never anticipating or participating in them? This paper examines these questions with respect to the discovery and further characterization of endogenous retroviruses (ERVs) in vertebrate genomes.

## 2. The Discovery of ERVs and the Emergence of the Endogenization-Amplification Theory

ERV papers frequently open by stating that about eight percent of the human genome is composed of sequences of retroviral origin. The prevailing view, which will here be called the endogenization-amplification theory, is that retroviruses have on many occasions inserted their genetic material in the germline DNA of host organisms. The integrated sequences, now transmitted vertically in a Mendelian fashion, may for a time have retained their ability to be transcribed and reintegrated elsewhere in the host genome, leading to their multiplication, or amplification, until they lost these abilities through mutation. This section will briefly outline the history of ERV research and the emergence of this theory of endogenization and amplification.

### 2.1. The Provirus Hypothesis

Since at least the 1940s, the term ‘provirus’ was used for (potentially oncogenic) particles that are normally transmitted by heredity, but are also capable of horizontal infection [4]. Yet it was not until 1964 that Howard Temin proposed the DNA provirus hypothesis, which states that the RNA of the Rous Sarcoma Virus acts as a template for the synthesis of DNA (the provirus), which is integrated in the host genome and in turn provides a template for new viral RNA [5]. Initially, this hypothesis was mainly supported by experiments showing that the inhibition of DNA synthesis obstructed the production of new Rous Sarcoma Virus [6]. Additionally, it was found that chicken cells infected by Rous Sarcoma Virus contained more DNA that is homologous with the RNA of the virus than non-infected cells, which was interpreted as evidence that the provirus was stably integrated in the nuclear DNA of the infected cells [7].

In Lakatosian terms, one could say that the provirus hypothesis functioned as a mini-research programme during the 1960s and 1970s, with its hard core being the thesis that certain RNA viruses reverse-transcribe their genetic material into the DNA of the host as part of their replication cycle. The positive heuristic encouraged proponents of the programme to look for evidence that viral sequences were indeed integrated into the host genome, and to identify the molecules responsible for reverse-transcription and integration.

That integration in the genome does occur was illustrated first for DNA viruses [8], and later for the Rous Sarcoma Virus [9]. The most important victory of the programme, however, came when RNA-dependent DNA Polymerase, predicted to exist years earlier [10] (p. 1090), was discovered in 1970 [11,12]. The enzyme, now known as reverse transcriptase, was soon shown to be widespread among RNA tumor viruses [13] and came to be recognized as a defining characteristic of retroviruses [14].

### 2.2. Endogenization and Phylogenetics

At around the same time, it was found that viral sequences were also present in uninfected chicken cells [15,16]. Moreover, Avian Tumor Viruses could be obtained from uninfected chicken cells [17] and Murine Leukemia Viruses from uninfected mice cells [18,19], suggesting that viral genomes were endogenous to these organisms.

If proviruses are endogenously present in the genomes of organisms, this raises the question of how long these sequences are maintained. Are they preserved across different species, or even higher taxa? When this question was answered in the affirmative, endogenous viral sequences (hereafter called endogenous retroviruses, or ERVs) immediately gained relevance for evolutionary biology. Soon, the earliest studies were published in which the homology between ERVs of different species were compared to the relatedness of these species. For instance, Kang and Temin [20] attempted to hybridize the RNA of Rous-associated virus-O (derived from chicken cells) to the DNA of uninfected cells of various bird species (DNA sequencing was still in its infancy at this time). They found that the Rous-associated virus-O RNA hybridized 55% to chicken DNA, 20% to pheasant DNA, 15% to Japanese quail DNA, 10% to turkey DNA, and <1% to duck DNA (they stated that these percentages correlate well with evolutionary distances from chicken, but the current understanding is that Japanese quail are closest to chicken, while pheasant and turkey are equidistant [21]). A similar but more extensive analysis, involving various primate species, was performed by Benveniste and Todaro [22]. They found that type C viral sequences were present in Old World monkeys, the great apes, and humans, and therefore concluded that these sequences must have been present in their common ancestor. They also found that the degree of homology between the baboon type C virus and sequences from the other species reflected their reputed phylogenetic relatedness.

In another study the same authors found that endogenous type C virus was present in four cat species, including the domestic cat, but was absent in many other *Felidae*, mink, and dog [23]. They concluded that at some point after the divergence between Old World monkeys and great apes, a type C virus of primate origin must have infected the common ancestor of the four cat species and inserted itself in the germline DNA. A similar study concluded that a murine virus invaded the pig genome between 10 and 3 Mya [24]. In other words, occasionally exogenous viruses endogenize, and the origin of the virus and the approximate timing of endogenization can be inferred by combining sequence similarities and independently derived phylogenies.

Note that the endogenization interpretation of Benveniste and Todaro was reached by assuming common ancestry of the species concerned and by employing two principles that are part of the standard heuristic toolbox of phylogenetics: (1) the assumption that the degree of sequence similarity reflects the degree of relatedness and (2) the criterion of parsimony. Other interpretations are possible, but these would either be non-evolutionary or would go against the heuristic of the evolutionary research programme. For instance, the type C virus could have been present in the common ancestor of cats and primates and have been lost in all lineages except primates and the one leading to these four feline species—but the loss of the provirus in many independent lineages would be far less parsimonious than a single horizontal transfer event. The endogenization interpretation of this and similar data must therefore be regarded as a result of the evolutionary research programme and its heuristic. Therefore, predictions of the endogenization hypothesis count as predictions of the overarching evolutionary programme.

One such prediction, that germline infection and subsequent Mendelian inheritance of retroviruses is at least possible, was investigated by Jaenisch [25]. He infected mouse embryos at the 4–8 cell stage with Moloney leukemia virus and out of 45 births he obtained three virus-positive animals, one of which was male. This male was used in backcrosses and sired 160 offspring, eight of which carried the viral genome in their DNA. In subsequent generations the virus was inherited according to Mendelian proportions. It should be noted that prior to infection the embryos were treated with pronase to remove the zona pellucida [26], which would normally protect the embryo from viral infection [27]. This would not occur in vivo. Also, being a leukemia virus, this particular ERV caused leukemia in the infected offspring and was therefore maladaptive. Nonetheless, the results were encouraging and later research has shown that new proviruses can be acquired by infection of the oocyte, before fertilization and the formation of the zona pellucida [28].

### 2.3. The Endogenization-Amplification Theory

Early on it was known that animal cells contain multiple copies of the same provirus (with some sequence divergence). Benveniste and Todaro [29] found 5 to 15 copies of C-type virus per haploid genome in various mammals. A decade later, BALB/c mice were known to possess 100–200 copies of VL30 [30]. By the early 21st century, the largest human ERV family (HERV-H) consisted of 1306 known copies [31].

These facts, combined with the finding that ERVs can be multiplied in the germline [32], led to the now dominant view on the origin of endogenous retroviral sequences. This view, here called the endogenization-amplification theory (EAT), is that exogenous retroviruses have on various occasions endogenized in animal genomes, with subsequent amplification by reintegration of copies [33]. This process has created ERV families ranging from a few to over a thousand loci in modern genomes.

## 3. The Heuristic of the Evolutionary Research Programme as Evident in ERV Research

As stated above, the negative heuristic of a research programme forbids any changes to the hard core. The positive heuristic is a programme’s problem-solving machinery. It is a somewhat malleable and evolving set of tools, principles, and suggestions on how to modify the protective belt [3] (p. 50). It tells researchers how to draw consequences from data [34] (p. 59), which questions to ask and how to answer them. The positive heuristic even provides the very vocabulary by which the data is simultaneously described and interpreted.

In the case of the evolutionary programme (and its subfield of phylogenetics in particular), the negative heuristic blocks researchers from accepting any proposition that contradicts large-scale common descent. They would not, for instance, accept an independent origin for mice and felines.

The central objective of the positive heuristic of phylogenetics is to classify reproducing entities (genes, viruses, organisms) according to their patterns of descent. The typical output is a phylogenetic tree showing the relationships among these entities. Because improbable events, such as arriving at a particular DNA sequence, are unlikely to occur multiple times, the most important, though not unbreakable, principle in constructing phylogenies is that the degree of similarity reflects the degree of relatedness. Criteria for judging phylogenetic trees are usually either maximum parsimony (the best tree is the one which requires fewest mutations or other events) or maximum likelihood (the best tree postulates the most likely series of events), depending on the school of thought. Thus, the positive heuristic bids researchers to compile large datasets of traits of the reproducing entities of interest, and apply algorithms to find the best tree, or to use an already established tree to reconstruct ancestral states, date last common ancestors, pinpoint the phylogenetic position of particular events, et cetera.

The positive heuristic of phylogenetics can be clearly seen at work with respect to ERVs. A very basic example of a phylogenetic inference is provided by Steinhuber et al. [35], who found that Old World monkeys possess a few copies of human endogenous retrovirus-K (HERV-K), whereas New World monkeys lack HERV-K, and duly inferred that HERV-K must have entered the human lineage after the split with the New World monkeys but before the split with the Old World monkeys. Also, since humans possess many more copies than Old World monkeys, they concluded that the HERV-K family must have greatly expanded after the human lineage split from Old World monkeys. Similar examples of the straightforward application of the phylogenetic heuristic are found in Shih et al. [36], who inferred that several ERV subfamilies must have been present in the common ancestor of Old World monkeys and apes, and in Goodchild et al. [37], who determined when individual HERV-H elements were integrated based on their phylogenetic distribution among humans, apes, and Old World monkeys. Examples of how the heuristic of the evolutionary programme handles more challenging data will be explored below.

## 4. Appraising the Evolutionary Research Programme

As stated, under MSRP the primary criterion for progress is that the research programme is supported by novel facts. I will here use the definition of ‘novelty’ as proposed by Worrall [34] and amended by Murphy: “A fact is novel if it is not used in the construction of the theory T that it is taken to confirm. A fact not used in the construction of a theory is one whose existence, relevance to T, or interpretability in light of T is first documented after T is proposed.” [38] (p. 68). This means that prediction, in a strictly temporal sense, is not a hard requirement—a programme is also supported by evidence that is postdicted in an appropriate way. I will here also accept ‘unanticipated corroboration’ by data that may not have been anticipated at the outset, but which can, in the light of some other new information, be shown to parsimoniously follow from the programme. An example in the current case study concerns target site duplications flanking ERV integrations (see Section 6).

The primary symptom for degeneration of the programme is the overutilization of ad hoc solutions for anomalies. Ad hoc maneuvers do not lead to novel predictions, or lead to predictions that are never corroborated, or disrupt progress by going against the grain of the programme’s positive heuristic [3] (p. 95). Note that anomalies will always abound—what counts is how they are dealt with. They should be explained in such a way that they lead to the anticipation of novel facts, and in accordance with the positive heuristic.

The following sections will apply these criteria to appraise how well ERV researchers have interpreted their findings within the broader evolutionary framework.

## 5. Orthologous ERVs

If ERVs have been endogenized and amplified over evolutionary history, as claimed by EAT, and if much of this evolutionary history is shared between different species, as claimed by the thesis of common descent, then we should expect that closely related species share the same ERV elements at orthologous loci. Moreover, the number of shared elements should reflect the degree of relatedness. For instance, all ERVs that integrated in the human genome after the gibbon lineage branched off but before the split with the chimpanzee, should be shared with chimpanzees (aside from deletions) but not gibbons.

This is indeed what is observed. Grandi et al. [39] searched the genomes of twelve non-human primates for orthologs of 211 human ERV-W elements. They identified 205 ERV-Ws in orthologous loci in chimpanzees, 207 in gorillas, 205 in orangutans, 190 in gibbons and 131 in rhesus macaques. Such large numbers of retroviral-like sequences in similar locations certainly fit expectations. The numbers of shared elements also cohere with the accepted phylogeny, although it must be hypothesized that chimpanzees lost a few more elements than gorillas and orangutans. All in all, this data strongly corroborates the evolutionary research programme.

It could be countered that ERVs represent just another instance of hierarchically distributed traits among organisms. That organisms can be classified in a nested hierarchy based on their similarities was already known to Linnaeus in the 18th century. It was also known to Darwin, for whom it belonged to the explananda of his theory [40] (Chapter 13). Since this nested hierarchy was used in the construction of the theory of evolution, by the criteria of MSRP it cannot be used a second time, in support of the theory. The appeal to shared ERVs as evidence for common descent could therefore be seen as a ‘more of the same’ argument. One more class of hierarchically distributed traits provides no support for common descent, any more than yet another reiteration of Pavlov’s experiment (but with a different kind of food, say) would increase our confidence in classical conditioning.

However, ERVs differ in one important respect from other shared traits. If interpreted correctly, endogenous retroviral-like sequences result from integration *events*. This means that they more directly attest to the *common history* of different species than other shared characteristics. Of course, this line of reasoning hinges on the premise that ERVs have indeed integrated. Support for this premise is found in the sequences within and surrounding ERVs, particularly target site duplications and long terminal repeats.

## 6. Target Site Duplications

During the late 1970s and early 1980s, researchers set out to map the genomes of retroviruses and their integrated proviruses. Among other things, it was found that the integration mechanism duplicates a short stretch of host DNA, resulting in direct repeats at both ends of the provirus [41,42,43]. This duplication arises because integrase makes a staggered cut in the host DNA to insert the viral DNA [44]. The duplicated segments are called target site duplications (TSDs). They are usually about 4 to 6 bp long, but sometimes much longer [45,46].

The discovery of TSDs created an unforeseen testcase for EAT. If retrovirus-like sequences really are endogenized retroviruses, then they should carry these marks of integration. Barring deletions or as yet unknown integration mechanisms, EAT predicts that all true ERVs sport target site duplications. This prediction appears to have come true, as the literature frequently refers to TSDs in the context of ERVs. For instance, the HERV-Ws discussed in the previous section, are generally flanked by 4-bp TSDs [47], unless they are amplified by the LINE-1 machinery, in which case their TSDs vary from 5 to 15 bp [48].

This evidence from TSDs falls in the ‘unanticipated corroboration’ category. Their existence was not anticipated when EAT emerged during the 1970s, but once the viral integration mechanism was sufficiently understood, it followed that endogenization implies the presence of TSDs. Consequently, TSDs represent a class of true ‘novel facts’ supporting EAT. This argument could be bolstered (or weakened) by a systematic investigation into the presence (or absence) of TSDs flanking ERVs.

## 7. The Divergence of Long Terminal Repeats

At around the same time, it was also revealed that proviruses contain direct repeat sequences, usually several hundred nucleotides long, at their 5′ and 3′ ends [49,50]. These sequences came to be known as long terminal repeats (LTRs). LTRs contain various regulatory sequences that are required for the replication of the virus, such as promotors, enhancers, and polyadenylation signals.

Significantly, the LTRs are identical upon integration and then diverge as the host genome incurs mutations [51]. This feature of LTRs, combined with EAT and the axiom of common descent, has several implications. First, to the extent that mutations in LTRs are selectively neutral, the degree of divergence between the two LTRs of an ERV could serve as a molecular clock dating the integration event. This approach has been used to date the insertion of retrotransposons, which also have LTRs [52]. Molecular clock estimates based on LTR divergence should correspond to the predicted ages of ERV elements based on their phylogenetic distribution.

A second, more intriguing, prediction is that under normal circumstances the 5′ and 3′ LTRs should have their own, independent gene trees. Specifically, the orthologous 5′ LTRs (or, by the same token, the orthologous 3′ LTRs) of *different* species sharing the same ERVs should be more similar to each other than the 5′ LTRs and 3′ LTRs within the *same* species. After all, the 5′ and 3′ LTRs have been diverging since the integration event, while the orthologous 5′ LTRs of two related species have only parted ways at the more recent speciation event.

### 7.1. LTR Divergence Compared to Phylogenetic Distribution

A modest initial test of the first prediction concerns two full-size ERVs in chimps, RTVL-1a and RTVL-1b, both located in the haptoglobin gene cluster. Maeda and Kim [53] determined that the LTRs of RTVL-1a differ by 10.1%, while those of RTVL-1b differ by 14.3%. They also found that the *pol* gene of RTVL-1a, which must have been functional at the time of integration, has incurred eight debilitating frameshift mutations, while the *pol* gene of RTVL-1b has incurred twelve. They therefore concluded that the RTVL-1b element is older. However, Shih et al. [36] reported a broader phylogenetic distribution for RTVL-1a, which is present in baboon, than for RTVL-1b, which is absent in baboon. So this first result contradicted phylogenetic expectations. The negative heuristic instructs us to explain this discrepancy without affecting the hard core of the research programme. One possible explanation would be that RTVL-1b originally had a broader phylogenetic distribution but was lost in the baboon lineage. A study of the haptoglobin gene cluster in rhesus monkeys has shown that this region is particularly susceptible to unequal crossovers [54], so the loss hypothesis is not entirely ad hoc.

More reassuring results were obtained by Barbulescu et al. [55] (p. 865), who listed eight uniquely human ERV-K elements along with two that are shared with great apes. The two shared ERVs had far more divergent LTRs than the uniquely human ERVs, consistent with phylogenetic expectations.

### 7.2. Independent Gene Trees of 5′ and 3′ LTRs

**Figure 2:**
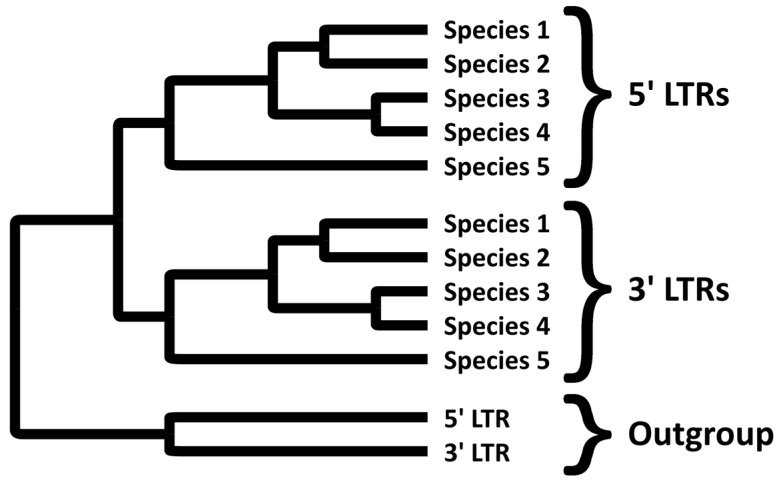
An ideal phylogenetic tree of LTRs of an orthologous ERV in five species. When the ERV integrates in the common ancestor, the 5′ and 3′ LTRs are identical. They then drift apart as they incur mutations. The two LTRs each keep an independent record of subsequent speciation events. All 5′ LTRs have been diverging from all 3′ LTRs since integration, whereas the 5′ LTRs amongst themselves have only been diverging since the various speciation events. All 5′ LTRs should thus clade together, as should all 3′ LTRs. The outgroup consists of a related ERV at another locus.

The thesis that similar retroviral-like sequences at identical loci in different species trace their origin to a retroviral integration in the common ancestor of these species, leads to a second prediction. This second prediction is that, all else being equal, the 5′ and 3′ LTRs should have their own, independent gene trees showing the order of speciation events that have occurred subsequent to integration (Figure 2). Notice, however, the ‘all else being equal’ clause. A topology such as in Figure 2 is only predicted if it is assumed that the two LTRs evolve independently of each other, they evolve at similar rates, there are no rearrangement events, and mutations in these sequences are selectively neutral [56]. Additionally, sufficient time must elapse between integration and the first speciation event for the LTRs to accumulate enough differences to form two clearly distinct clusters [57]. All of these assumptions belong to the protective belt of the programme. If the sequence data does not yield the expected phylogeny, one or more of these assumptions would take the blame for the failed prediction, rather than the core notion of common descent.

A case in point is the analysis of Johnson and Coffin [56]. They constructed phylogenies based on LTR sequences of six human ERVs (HERVs) and their orthologues in other primate species. Two of these (HERV-K(HML6.17) and RTVL-Ha) produced trees that mostly resembled the predicted topology, except for some peculiar arrangements within the Hominoidea. The other four ERVs deviated from the predicted pattern to varying degrees. It is of interest to see how the authors explain these discrepancies.

1. The most parsimonious tree for HERV-K18 contained an anomalous clade of the gorilla 5′ and 3′ LTR. The authors offered various solutions. In either the gorilla lineage or the human/chimp lineage, the two LTRs could have homogenized each other through non-allelic gene conversion (NAGC), or in one of the lineages the ERV was mostly replaced by a very similar provirus by ectopic recombination. They even contemplate the possibility that closely allied but different ERVs integrated at identical loci in different species, but they deem this highly unlikely. Unfortunately, they offer no independent way to test any of these explanations.
2. HERV-K(C4) produced the predicted tree topology; however, this tree only contains three taxa: humans, orangutans, and African green monkeys. Notably, the ERV was not detected in chimpanzees and gorillas. Dangel et al. [58] propose that the ERV never drifted to fixation. Instead, the locus remained polymorphic, with both the ERV and the original, pre-integration site coexisting side by side in primate populations for tens of millions of years, until the ERV was finally lost in chimpanzees and gorillas. Johnson and Coffin, on the other hand, side with Klein et al. [59], who argue for frequent homogenization in this region. This must have happened independently in chimpanzees and gorillas.
3. In the RTVL-1a tree, the gibbon 5′ LTR anomalously clades with the 3′ LTRs. Johnson and Coffin identify the substitutions that are responsible for this and conclude that part of the gibbon 3′ LTR was transferred to the 5′ LTR through gene conversion. Removing the gibbon 5′ LTR from the analysis restores a mostly correct topology.
4. Lastly, the RTVL-Hb tree in no way resembles the predicted phylogeny. The authors suggest that the RTVL-Hb sequences may frequently recombine with other RTVL-H loci. In support, they note that RTVL-H is a very large ERV family with over a thousand members throughout the genome, increasing the chances for non-allelic recombination. This line of reasoning could be criticized for explaining too much, as recombination with its multitudinous family members does not seem to have affected RTVL-Ha, which was one of the two well-behaved ERVs in the Johnson and Coffin study. Moreover, they produce a phylogenetic tree containing LTRs of four members of the RTVL family (including RTVL-Hb) and draw attention to the fact that LTRs belonging to the same provirus neatly group together. This is inconsistent with frequent ectopic recombination within this family.

So most loci do not produce the predicted tree topology, which means that, in line with MSRP, one of the assumptions contained in the ‘all else being equal’ clause must give way. In this case, the assumption that is sacrificed is that LTRs evolve independently. It is replaced by the hypothesis that LTRs are occasionally homogenized by NAGC. In other words, gene conversion is an auxiliary hypothesis within the protective belt of the research programme. As stated above, such changes to the protective belt are assessed by whether they lead to new predictions, and by the extent to which these predictions are corroborated. If the evidence for NAGC solely consists of its ability to explain anomalous tree topologies, then the appeal to conversion is a degenerative move, but if it leads to the discovery of novel facts, or at least receives unanticipated corroboration, then there is empirical progress within the programme.

Fortunately, gene conversion is not just a theoretical construct, but a mechanism that has been known and studied for several decades. Although our understanding is far from complete [60], research in this area has revealed ways to test the feasibility of NAGC as an explanation for discordant phylogenies. A non-specific (i.e., not locus-specific) test is to examine whether the frequency at which NAGC is needed to explain phylogenetic patterns, correlates with the presence of conditions that are known to favor the occurrence of NAGC. A good example of this is reported by Kijima and Innan [61], who looked for full-length LTR retrotransposons shared between two rodents (mice and rats), as well as between two primates (humans and rhesus macaques). Assuming that these shared sequences were inherited from a common ancestor, they determined how often gene conversion was needed to account for the sequence data. They found that NAGC has to be much more prevalent in the rodents than in the primates. This finding is consistent with independent evidence that the mouse genome is more amenable to gene conversion events than the human genome. The molecular basis for this is that the ‘minimal efficient processing segment’ in mice is shorter than in humans. It was estimated that efficient recombination in mice cells requires at least 134 to 232 bp of uninterrupted homology [62], while in humans this may be approximately 337 to 456 bp [63]. There are also locus-specific ways to assess the validity of the NAGC auxiliary hypothesis, as will be discussed below.

### 7.3. Combining LTR Phylogenies and LTR Divergence Dating

Gene conversion between the two LTRs of an ERV will not only disarrange tree topologies, it also affects the LTR divergence approach for dating ERV integrations [61]. Since NAGC erases some of the accumulated differences between the two LTRs, it makes the ERV appear younger. NAGC could therefore be invoked to explain the failure of both predictions mentioned above: it could explain why some ERVs produce molecular clock dates that are too young considering their wide phylogenetic distributions, and it could explain why 5′ and 3′ LTRs do not always form two separate clades in LTR phylogenies.

It may seem that NAGC renders the evolutionary research programme as a whole less testable; however, testability may be restored by looking for independent, locus-specific evidence for NAGC, and by combining the phylogenetic and the molecular clock approaches. For those ERVs whose phylogenies and sequence data show no sign of conversion events, we can more confidently predict that their ages based on LTR divergence should agree with their phylogenetic distribution (although there could still be other explanations, besides NAGC, if the prediction fails), but where tree topologies do indicate conversion between the 5′ and 3′ LTRs, any molecular clock discrepancies are expected to be tilted towards the ‘too young’ side.

The most useful study in this area was performed by Hughes and Coffin [57]. They subjected human ERVs and their primate orthologues to both phylogenetic and molecular clock analyses. I will summarize their results and interpret them through the spectacles of MSRP.

Of the fifteen ERVs Hughes and Coffin analyzed (Table 1), nine produced phylogenies that mostly or perfectly fit the predicted topology. This in itself (9 out of 15) is a good result. The six remaining phylogenies contained anomalies that warranted a deeper look into the possibility of NAGC. Of these six ERVs, two gave molecular clock estimates that agreed with their species distribution, but the other four gave age estimates that were too young—as expected if NAGC occurred. For two of those, the authors reported independent corroborating evidence.

There are various ways to detect gene conversion in a sequence [64]. One of these, the co-double method [65], was employed by Hughes and Coffin to demonstrate conversion at the HERV-K(II) locus. This is a statistical test that looks at the frequency of so-called ‘co-doubles’, which are substitutions shared between two duplicated stretches of DNA (in this case, the two LTRs) within the same species. For HERV-K(II), there was statistically significant evidence for gene conversion in all four species.

Persuasive independent evidence for NAGC was also found for 20q11, although by a different method. At that locus, the authors identified a 400 to 500 bp stretch of DNA that was transferred from the 3′ to the 5′ LTR in the ancestor of chimpanzees and bonobos. Removing that part of the sequence from the analysis improved the tree topology. However, eliminating chimpanzees and bonobos from the molecular clock analysis still resulted in an integration date several million years too young compared to the phylogenetic distribution.

None of the other loci with discordant phylogenies showed evidence for NAGC. In fact, each of these ERVs has its own unique story. The authors reject conversion in the case of 9Q34.3, instead opting for the hypothesis that the ERV integrated just before the orangutan lineage split off from the human/African ape lineage. No independent evidence is offered, other than the fact that this could explain the unexpected position of the orangutan LTRs, whereas NAGC could not. For 6p21 they also rejected NAGC. They propose that part of an ancient ERV was mostly replaced by another ERV element by homologous recombination in the common ancestor of humans and African apes. They cite various lines of evidence for this hypothesis, such as a higher concentration of substitutions at the outer edges of the LTRs, which may be the remnants of the original ERV, and a temporary rise of the inferred substitution rate around the time of the replacement. Yet another explanation is proposed for the discordant phylogeny of 10p14: the same substitutions have occurred independently in different lineages (homoplasy). This is supported by the fact that most of the homoplastic substitutions occurred in CpG sites, which are known to be mutational hotspots [66]. Finally, for the 12q24 locus, Hughes and Coffin suggest either NAGC or the independent divergence of one of the human LTRs. Since both chimpanzees and gorillas have apparently lost their 3′ LTR, there is little data to support either of these hypotheses.

Turning to the nine ERVs with predicted tree topologies, we see that five generated molecular clock estimates which are consistent with their species distribution (Table 1). The other four were dated too old, meaning that they integrated so long ago that they should have been present in more species. For instance, 19p13.11B was estimated to have integrated at least 26.4 million years ago. This was before the Old World monkeys and gibbons branched off from the human lineage (according to the dates they used). Yet, 19p13.11B was not detected in Old World monkeys and gibbons. Again, there are several ways in which the protective belt can accommodate these anomalies. Perhaps the ERVs *are* present in these species, but they were simply not detected. This is not unconceivable, because the primer sequences used for PCR amplification were based on the human flanking sequence, so they may not have been conserved in distant species. Or maybe the ERVs were lost in certain species, narrowing their phylogenetic distribution. Or perhaps the molecular clocks do not run at a constant rate—an elevated mutation rate between integration of the ERV element and the first speciation event would lead to an age overestimation. These are all real possibilities; however, under MSRP they are regarded as ad hoc solutions, unless they can be backed up by independent evidence. The temptation exists to opportunistically appeal to complicating factors when there are anomalies to be resolved, while not even mentioning these complications when their services are not needed. To resist the selective appeal to auxiliary hypotheses, we need evidence that discriminates between those instances when they do apply, and those when they do not.

## 8. Conclusions

Employing the criteria of MSRP, this paper appraises the evolutionary research programme as it has guided half a century of ERV research. Based on the issues here discussed, it is concluded that the programme has been empirically progressive.

As was seen, the core notion of common descent and the heuristic rule that similarity implies common descent, have led to the emergence of the endogenization-amplification theory, which has become the dominant view of the origin of genomic retroviral-like sequences. The finding that large numbers of ERV elements are shared at orthologous loci in different species, strongly coheres with the combination of common descent and EAT. Furthermore, this view received unanticipated corroboration from the discovery of TSDs flanking many ERVs.

Regarding the divergence of LTRs, the programme was only mildly progressive. The prediction that the degree of divergence between the two LTRs should agree with the phylogenetic age of the ERV held true for some ERVs but not for others. A second prediction, that the two LTRs should produce two independent gene trees, consistent with accepted phylogeny, was more successful. Most of the loci investigated by Hughes and Coffin [57] produced largely correct phylogenies. Moreover, the majority of the discordant trees could be explained by auxiliary hypotheses that enjoy independent support. Yet, the large number of potential ‘escapes’ does give pause. A critical observer might charge that, regardless of the data, there will always be *some* rationalization that happens to align with a piece of independent evidence. That is why, under MSRP, an auxiliary hypothesis must be supported by *novel* facts, which are found after the hypothesis is proposed.

Due to the magnitude of the subject, this analysis has been far from exhaustive. It did not include some relevant topics, such as solo LTR formation [67] and the currently ongoing ERV invasion in koalas [68]. Of particular interest for future study would be the programme’s ability to cope with the shifting status of ERVs from potentially selfish genetic elements [69,70] to crucial contributors to the host’s survival and reproduction [71,72,73].

## Figures and Tables

**Table 1:** ERVs at fifteen loci analyzed by Hughes and Coffin [57]. Column 3 is based on the data reported in Table 1 of their paper. They assumed the gorilla lineage split off from the human/chimpanzee lineage 7 million years ago. Current estimates give an earlier date for this branching event, which could change some of the assessments in this column. Columns 4 and 5 summarize the authors’ proposed explanations for the discordant tree topologies and the independent evidence they found for these hypotheses.

Name/Locus	Predicted Tree Topology	Clock Age vs. Species Distribution	Auxiliary Hypothesis	Independent Evidence
1q23	Yes	Correct	-	-
3p25	Yes	Correct	-	-
11q12	Yes	Correct	-	-
19p13.11A	Yes	Correct	-	-
19q13.1	Yes	Correct	-	-
4q32	Yes	Too old	-	-
6p22	Yes	Too old	-	-
19p13.11B	Yes	Too old	-	-
22q11	Yes	Too old	-	-
10p14	No	Correct	Homoplasy	CpG mutation hotspots
12q24	No	Correct	NAGC or independent divergence of human 5′ LTR	None given
HERV-K(II)	No	Too young	NAGC in all species	Co-double method
6p21	No	Too young	Replacement of old ERV by new element	Most substitutions at ends of LTR sequences + seeming temporary rise of mutation rate
9q34.3	No	Too young	Integration just prior to speciation	None (or failure of the NAGC hypothesis)
20q11	No	Too young	NAGC in chimp/bonobo	Converted fragment identified in sequence
